# Biocontrol Efficacy of *Metschnikowia* spp. Yeasts in Organic Vineyards against Major Airborne Diseases of Table Grapes in the Field and in Postharvest

**DOI:** 10.3390/foods12183508

**Published:** 2023-09-21

**Authors:** Monia Federica Lombardo, Salvina Panebianco, Cristina Restuccia, Gabriella Cirvilleri

**Affiliations:** Di3A, Dipartimento di Agricoltura, Alimentazione e Ambiente, University of Catania, Via S. Sofia 100, 95123 Catania, Italy; monia.lombardo@phd.unict.it (M.F.L.); salvina.panebianco@phd.unict.it (S.P.); gcirvil@unict.it (G.C.)

**Keywords:** biocontrol yeasts, *Metschnikowia pulcherrima*, *Metschnikowia fructicola*, organic grape, *Erysiphe necator*, *Botrytis cinerea*

## Abstract

The aim of this work was to evaluate the efficacy of two antagonistic yeasts, *Metschnikowia pulcherrima* strain MPR3 and *M. fructicola* strain NRRL Y-27328 (commercial product NOLI), applied in addition to the “on-farm biological treatments” (BIO) carried out during the production season, for the containment of powdery mildew and grey mould diseases on organic table grapes ‘Italia’. The yeast strains were applied in the field three times, and their efficacy was evaluated during the production season and under postharvest conditions. Overall, *M. pulcherrima* MPR3 combined with BIO treatments reduced disease incidence caused by *Erysiphe necator* and disease incidence and severity caused by *Botrytis cinerea* with values between 67.8% and 86.2%, showing higher efficacy than BIO treatments applied alone and in combination with NOLI. Field treatments based on BIO+MPR3 maintained their performance also during fruit storage, protecting grape berries from grey mould development to a greater extent than the other treatments (disease reduction of about 98%). Thus, the presence of *M. pulcherrima* MPR3 seems to improve disease management both in the field and in postharvest environments, without negative impacts on grape microbial communities. These findings highlight the potential of *M. pulcherrima* MPR3 as a promising alternative strategy for disease control in organic vineyards and in postharvest, providing sustainable solutions to improve food quality and safety.

## 1. Introduction

The grapevine (*Vitis vinifera* L.) is one of the most widely cultivated plant species of agricultural interest, and table grapes are considered to be one of the most popular fruit crops in the world. The world production of table grapes is about 27,300,000 tons [1], with an annual increase over the last decade of about 5–7%. Europe holds second place in the world in terms of cultivated area (3,600,000 ha) and production (34,000,000 quintals/year). Italy is the leading table-grape producer in Europe and is the main European exporting country, not so much in terms of cultivated area (47,583 ha) as in terms of quantity produced (10,103,977 quintals in 2022; ISTAT, http://dati.istat.it/Index.aspx?QueryId=33706&lang=en, accessed on 21 April 2023). In Sicily, the most cultivated areas are located in west-central and eastern Sicily, where mild winters and warm summers allow both production diversification and an extensive harvesting calendar. In recent years, the growing consumer demand for safer, higher-quality and healthier foods has led to a spread adoption of organic agriculture. As part of this global trend, organic grapevines in Europe are grown on about 4,000,000 hectares, with Spain, France and Italy ranking first with 350,000 hectares (73.5% of the total), with slight differences among the three countries [2]. In Italy, the organic grapevine area reached 109,000 hectares in 2019, and 2% of this area (2281 hectares) is for table-grape production [3], mainly distributed in Apulia (74%) and Sicily (18%) [4].

Powdery mildew, grey mould and downy mildew, caused by *Erysiphe necator*, *Botrytis cinerea* and *Plasmopara viticola*, respectively, represent the most important grapevine diseases posing a serious threat to yield and quality worldwide, including in the Mediterranean viticultural regions. In southern Italy, *E. necator* is an important limiting factor in grape production, along with *B. cinerea*, and both are more prevalent than *P. viticola*, which instead poses a major challenge to grape production in northern Italy. The defence of vineyards under organic regimes currently depends on the use of copper and sulphur, which are the pivotal active ingredients against powdery mildew, grey mould and downy mildew. The negative effects on biodiversity and human health induced by their heavy use are widely demonstrated, and in particular, the use of copper is increasingly carefully regulated and reduced [5,6]. New products as an alternative to copper and more generally alternatives to chemicals are being tested on a laboratory scale, in controlled environments and in the field [7,8]. Within them, products based on biocontrol agents (BCAs) appear particularly promising for their multiple positive mechanisms of action against phytopathogens on numerous crops.

Among BCAs, several yeasts have demonstrated antagonistic activity against different fungal pathogens of fruits and vegetables [9,10], with several mechanisms of action [11,12,13,14,15,16]. Among the yeast species, *Metschnikowia* spp., *Aureobasidium* spp. and *Debaryomyces* spp. [13,17] have already demonstrated their potential for the control of postharvest decay of fruits and vegetables. Their ability to easily colonize dry surfaces, to compete for space and nutrients and to secrete lytic enzymes, along with their simple nutrition requirements, are positive characteristics that may change across genera and species. Moreover, the mechanisms of action may vary depending on climatic and agronomic conditions encountered by BCAs in vineyards, thereby representing a key factor to the complete efficacy of yeast treatments.

*M. pulcherrima* is a relevant antagonistic yeast species that has been successfully employed for the biological control of fruit and vegetables [16,18,19,20,21], and the competition for nutrients, especially for iron, has been demonstrated to be the dominant mechanism of action [13,22]. *M. pulcherrima* MPR3 strain, in previous trials conducted in vitro and on a laboratory scale, showed good efficacy against *B. cinerea*, *Penicillium digitatum*, *P. italicum*, *Aspergillus flavus*, *Monilinia fructicola* and *M. fructigena* on numerous crops, including table grapes [13,16,17,23]. However, to the best of our knowledge, there is currently no study available on organic table grapes for disease management in the open field and in postharvest conditions by *Metschnikowia* spp. when applied during the growing season accordingly with different phenological stages. Karabulut et al. [24] reported the efficacy of *M. fructicola*, ethanol and sodium bicarbonate (SBC), alone or in combinations, applied to table grapes on vines 24 h before harvest against *B. cinerea*, *Alternaria* spp. or *A. niger* during storage. More recently, Agarbati et al. [25] focused on yeast treatments of wine grapes, comparing them with a commercial biological fungicide against *B. cinerea*, and investigated yeast survival and efficacy under postharvest commercial conditions for wine grapes.

Therefore, the aim of this study was to assess the effectiveness of *M. pulcherrima* strain MPR3 (part of the microbial collection of Di3A, University of Catania) and *M. fructicola* NRRL Y-27328 (commercial product NOLI) in managing powdery mildew and grey mould on Sicilian organic table grapes ‘Italia’ both in the field (i) and in postharvest conditions (ii), comparing their performance with the organic treatments currently employed in the vineyards (iii). Moreover, we investigated the effects of yeast treatments on cultivable carpospheric microorganisms and the survival of *Metschnikowia* spp. on the fruit surface at the end of the storage period.

## 2. Materials and Methods

### 2.1. Field Experiments

**Figure 1:**
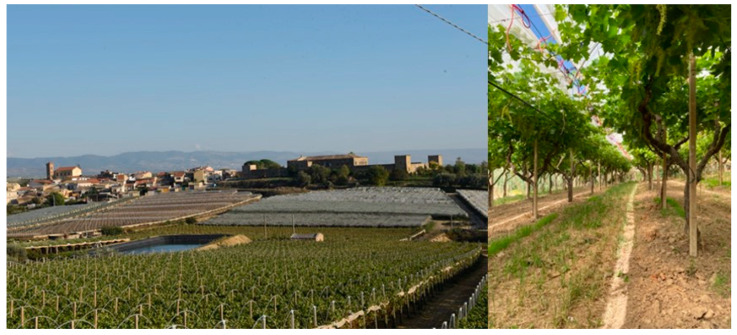
Experimental site in Mazzarrone, Catania province (Italy).

Field experiments were performed in duplicate during 2022 in two 10-year-old organic vineyards of *Vitis vinifera* L. on table grapes ‘Italia’ grafted on 140 Ru (Ruggeri) rootstock. The two field experiments were conducted in vineyards located in Mazzarrone (Catania province, Italy, 37°03′31″ N 14°33′49″ E) (Figure 1). The plants, covered with annual polyethylene plastic film, were spaced 2.8 m apart within the rows, with 2.8 m between the rows and intercropped with fava beans between the rows during winter. The vineyards were irrigated during summer using drip-line irrigation, which was also used for fertirrigation. During winter, organo-mineral fertilizer [Armonis, NPK (Mg-S) 4–8-10 (2–8), BioUnimer] was distributed at the rate of 500 kg ha^−1^ banded under the grapevine, according to the common practices for the cultivation area. During the production season, biological treatments for pest management allowed in organic production were applied. The applied treatments, in this study referred to as “on-farm biological treatments” (BIO), were based on the following commercial products: copper oxychloride (Cupravit^®^ 35W, Bayer Cropscience S.r.l., Milano, Italy, 1 kg ha^−1^, 8 applications); sulphur 80% (Tiovit^®^ Jet, Syngenta Italia S.p.a., Milano, Italy, 2 kg ha^−1^; 8 applications); *Bacillus pumilus* QST2808 (Sonata^®^, Bayer Cropscience S.r.l., Milano, Italy, 5 L ha^−1^; 6 applications); COS-OGA (Ibisco^®^, Gowan Italia S.rl., Faenza, Italy, 2 L ha^−1^; 5 applications); Spinosad (Laser^®^, Corteva Agriscience S.r.l., Cremona, Italy, 0.2 L ha^−1^; 3 applications); rock dust (Abomin, Abomin SP sl., Madrid, Spain, 2.5 kg ha^−1^; 3 applications).

In this study, treatments based on *M. pulcherrima* MPR3 (collection of Di3A, University of Catania) and *M. fructicola* strain NRRL Y-27328 (NOLI, Koppert Italia S.r.l., Bussolengo, Italy) were added to BIO treatments, and their efficacy was compared with BIO treatments and with untreated control.

The trials, based on four theses (control, BIO, BIO+NOLI and BIO+MPR3) were carried out following a randomized block experimental design with three replicates per treatment and six plants per replicate (plot), for a total of 18 plants per treatment. An untreated row was left between the different plots subjected to experimental treatments. BIO started on 26 April, when the plants were at the phenological stage of six or more leaves unfolded (budding in the initial phase) and with a shoot length of approximately 7 cm, and ended on 24 September. *M. pulcherrima* MPR3 (10^10^ cells/L, prepared as described in Parafati et al. [13]) and NOLI (*M. fructicola* strain NRRL Y-27328; 2 kg ha^−1^) were applied by using a 16 L knapsack sprayer with an electric motor and a head equipped with two 2 mm nozzles, distributing a volume of water of approximately 20 L of solution per plot. The experimental treatments with MPR3 and NOLI were applied on 19 June (fruit swelling), 23 July (beginning of veraison) and 14 September (full ripening). On these dates, BIO treatments were never applied.

### 2.2. Climatic Data

The meteorological parameters, i.e., mean temperature (°C), relative humidity (%) and rainfall (mm) were obtained from data provided by the weather stations in Mazzarrone (province of Catania), Servizio Informativo Agrometeorologico Siciliano (SIAS), Region of Sicily, Italy. These data were implemented with the phenological phases of vine budbreak, bloom and veraison.

### 2.3. Symptom Evaluation

The incidence and severity of powdery mildew (*E. necator*) and grey mould (*B. cinerea*) were assessed, in each plot of the two vineyards and for each monitoring time and repetition, by visual estimation of the number of affected bunches and affected area in each bunch. Powdery mildew infections were evaluated four times (30 July, 16 August, 2 September and 23 September 2022), whereas grey mould infections were evaluated three times (2 September, 23 September and 6 October 2022). Observations covered 25 bunches per plant for a total of 150 bunches per plot (6 plants/plot), and 450 bunches per thesis (3 plots/thesis) were assessed. Disease incidence (DI%) was calculated as the percentage of infected bunches out of the total number of examined bunches for each treatment by using the following formula:

(1) $$DI\left(\%\right)=\frac{\mathrm{Number}\;\mathrm{of}\;\mathrm{infected}\;\mathrm{bunches}}{\mathrm{Total}\;\mathrm{number}\;\mathrm{of}\;\mathrm{assessed}\;\mathrm{bunches}}\times 100$$

To calculate disease severity (DS), an empirical scale (0–3) of four grades was used, with class 0 showing no symptoms and class 3 representing the highest damage. In detail, the damage values for each class correspond to a percentage range of infections on bunches, where class 0 = 0%, class 1 = 1–25% of infected berries on a single bunch, class 2 = 25.1–50% of infected berries on a single bunch and class 3 > 50% of infected berries on a single bunch. The average class (weighted mean) value of examined bunches (=disease severity, DS 0–3) was evaluated for each treatment.

Moreover, the infection index (or McKinney’s index, I_MK_), which combines both the incidence and severity of the disease, was evaluated according to the following equation:

(2) $$I_{MK}=\sum \frac{d\times f}{N\times D}$$

where d is the category of disease class scored for the grape bunches, f is the disease frequency, N is the total number of examined bunches and D is the highest class of disease intensity that occurred on the empirical scale.

To quantify disease progress, the area under disease progress (AUDPC) was calculated by using the following formula:

(3) $$\mathrm{AUDPC}=\sum_{i=1}^{N_{i-1}}\frac{\left[(y_{i}+y_{i+1}){(t}_{i}+t_{i+1})\right]}{2}$$

where y_i_ is the infection index at the i^th^ evaluation, y_i+1_ is the infection index at the i^th + 1^ evaluation, t_i+1_ and t_i_ are the number of days between the two assessments.

### 2.4. Effect of Field Treatments on Postharvest Environments

Twenty days after the last field application of the antagonistic yeasts, representative grape bunches were collected to evaluate the ability of strains to protect fruits from grey mould caused by *B. cinerea* under postharvest condition. For this purpose, for each thesis (control, BIO, BIO+NOLI and BIO+MPR3), apparently healthy and undamaged grape bunches (18 bunches/treatment, 6 bunches/replicate) were randomly harvested, transported in the laboratory and placed in cardboard containers (three boxes/replicate, 9 boxes/treatment). Then, boxes were stored in a climatic cell (90% RH) at 5 ± 1 °C for 10 days (cold storage) and, subsequently, at 20 ± 1 °C for 4 days. At the end of the storage at low temperature (10 days) and after the whole storage period (14 days) (i.e., after 30 and 34 days from the last field application), disease incidence (DI), severity (DS) and the infection index (I_MK_) of grey mould caused by *B. cinerea* were evaluated, following the procedure described in Section 2.3.

### 2.5. Evaluation of Epiphytic Microbial Population Associated to Grape Berries

At the end of the whole storage period, representative grape samples were selected in order to assess the viable total fungal and bacterial populations, as well as the *Metschnikowia* spp. population. For each thesis, healthy and undamaged grape berries were randomly and aseptically selected from each of the three boxes containing the respective replicates. Then, 100 g of grape berries from each replicate was transferred into sterilized flasks containing 200 mL of sterile 0.1 M potassium phosphate buffer (PPB), shaken for 45 min at 200 rpm and then sonicated for 8 min in a sonication water bath (L & H Manufacturing Company, Kearny, NJ, USA). Appropriate 10-fold serial dilutions in 0.1 M PPB were spread on Wallerstein Laboratory (WL) nutrient agar (Merck KGaA, Darmstadt, Germany) and on potato dextrose agar (PDA) (Oxoid, Basingstoke, UK), both supplemented with 0.005% chloramphenicol (AppliChem GmbH, Darmstadt, Germany) to prevent bacterial growth and on nutrient agar (NA, Oxoid) supplemented with 0.005% cycloheximide (AppliChem GmbH, Darmstadt, Germany) to prevent fungal growth. The plates were incubated at 25 °C for 3–5 days, after which fungal, bacterial and *Metschnikowia* spp. colonies were counted on PDA, NA and WL, respectively. Data were calculated as CFU/gr and expressed as Log10 CFU/g. The data were average values of six replicates for each treatment. Based on the differential properties, WL made it possible to distinguish the red-appearing colonies of *Metschnikowia* spp. from other indigenous yeast species, although it was not possible to distinguish any indigenous *Metschnikowia* spp. strains from those sprayed with the treatments.

### 2.6. Statistical Analysis

Data from the field and postharvest experiments and microbial populations were analysed separately using the Minitab™ software, version 20.0. In detail, experimental data from field trials concerning disease incidence (DI), disease severity (DS) and McKinney’s index (I_MK_) were subjected to two-way analysis of variance (ANOVA). Statistical analysis was conducted by calculating F and the correspondent *p*-values to evaluate whether the effects of single factors (treatment, site and monitoring time) and their interactions were significant. The differences among treatment performances under postharvest conditions and their effects on microbial populations living on grape berries were evaluated through one-way ANOVA. In all experiments, before proceeding with analysis, arithmetic means of DI, DS, I_MK_ and microbial communities (CFU/g) were calculated by averaging the values of replicates for each treatment. Percentage data for DI and I_MK_ were transformed into arcsine (prior to statistical analysis). For all trials, post hoc comparisons of treatments were obtained using Fisher’s least significant difference test (LSD) at *p*-value < 0.05.

## 3. Results

### 3.1. Climatic Data

**Figure 2:**
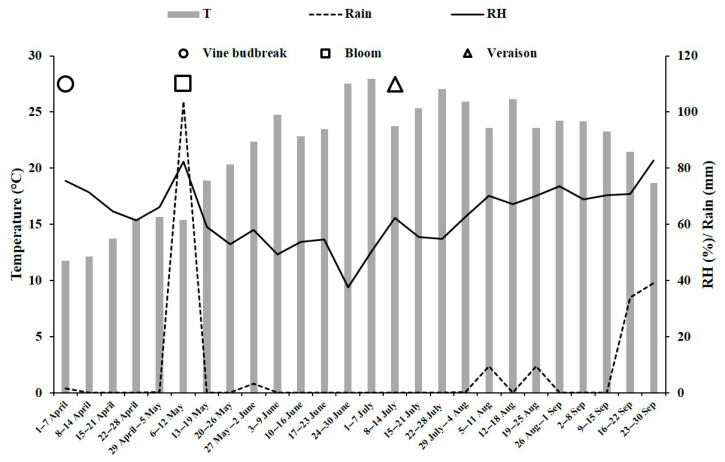
Climate data and main phenological stages of grapevine ‘Italia’ detected in the 2022 season from April to September by the weather station of Mazzarrone (Catania, Italy). Data are mean values of daily climatic surveys. T—temperature; RH—relative humidity.

Overall, climatic conditions detected in the 2022 season were not favourable to the development of downy mildew, powdery mildew and grey mould in table grapes ‘Italia’. In Figure 2, the mean weekly values of air temperature, relative humidity and rain are reported by averaging raw data recorded from 1 April to 30 September by the SIAS meteorological station in Mazzarrone (Catania, Italy). Moreover, in Figure 2, phenological stages of vine budbreak, bloom and veraison are indicated. Spring was characterized by high temperatures and drought, with the exception of abundant rainfall in mid-May, which resulted in a lower risk of infection. These disease-unfavourable conditions continued throughout the summer period until mid-September. The prolonged drought resulted in the use of drip-line irrigation, which did not, however, create a microclimate favourable to disease development.

### 3.2. Efficacy of Field Treatments against E. necator and B. cinerea

**Figure 3:**
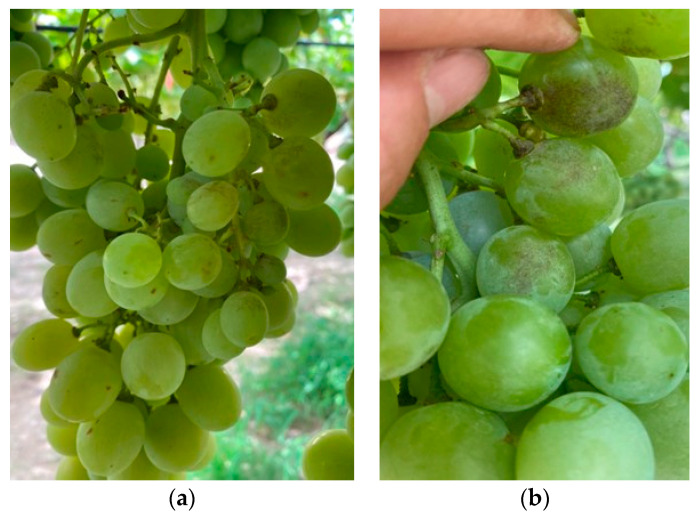
Powdery mildew symptoms caused by *Erisyphe necator* on bunches of table grape ‘Italia’ treated with *Meschnikowia pulcherrima* MPR3 (**a**) and *Meschnikowia fructicola* NRRL Y-27328 (NOLI) (**b**). Class of severity 1 (1–25%).

The trials were aimed at evaluating the efficacy of two antagonistic yeasts belonging to the genus *Meschnikowia*, *M. pulcherrima* strain MPR3 and *M. fructicola* strain NRRL Y-27328, in reducing powdery mildew and grey mould decay. These BCAs were applied on vines ‘Italia’ under an organic regime by performing three treatments (19 June 2022, 23 July 2022 and 14 September 2022) in addition to the organic treatments commonly carried out in the vineyards (BIO). The efficacy of *M. fructicola* NRRL Y-27328 and *M. pulcherrima* MPR3 against *E. necator* (Figure 3) was evaluated in the field over 45 days from 30 July to 23 September 2022. To evaluate the effects of the single factors (“treatment”, “site”, “monitoring time”) on disease parameters, experimental data were subjected to two-way analysis of variance. Effects of the single factors “treatment” and “monitoring time” were always significant for all the tested parameters (*p*-value ≤ 0.0001), whereas “site” effects were not significant (Table 1). Moreover, the interactions between factors were not significant on DI, DS and I_MK_ variables. Therefore, the treatments showed similar efficacy in the two different fields and over time. Based on the ANOVA results, data from the two field trials were averaged and reported for each monitoring time.

Post hoc analyses to establish the ranking of treatments’ effectiveness are reported in Table 2. As shown, significant differences in the values of DI, DS and I_MK_ were observed among different treatments over time. At the last monitoring time, treatment BIO+MPR3 exhibited the best performance, reducing the number of infected bunches by 86.2% if compared to the untreated control. Although to a lesser extent, BIO treatments, alone and in combination with NOLI, were also able to significantly reduce the powdery mildew decay if compared to the untreated control (DI reductions of about 48 and 52%, respectively). Similarly, all treatments significantly reduced DS and I_MK_ values if compared to the untreated control, showing values of reduction from 42.8% up to 73.5% at the last monitoring time.

The best results were achieved when BIO was integrated with NOLI or with MPR3. In fact, the DI and I_MK_ values were lower on bunches treated with BIO+MPR3 and BIO+NOLI and higher on BIO-treated grapes and on the untreated control (Table 2).

**Figure 4:**
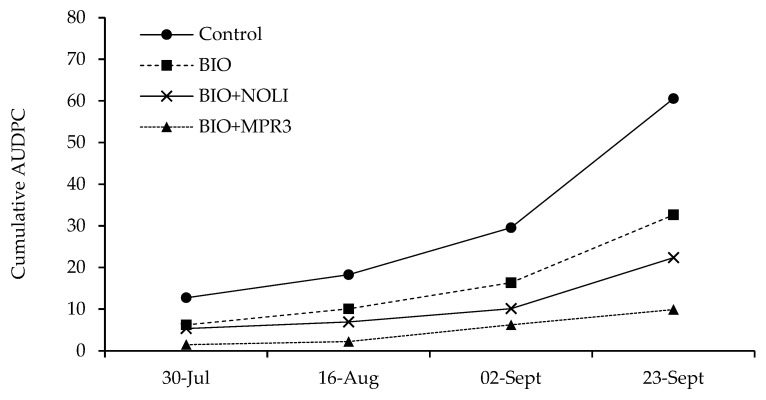
Area under disease progress curve (AUDPC) based on the McKinney’s index (%) of *Erysiphe necator* on treated grapes with different formulations.

The analysis of AUDPC from 30 July to 23 September (Figure 4) revealed more clearly that the increase in *E. necator* symptoms over time was strongly affected by treatments, confirming the previous results reported in Table 2. Powdery mildew progressed quickly in untreated bunches (control) and more slowly in BIO- and BIO+NOLI-treated bunches, reaching final AUDPC values of 60.6, 32.6 and 22.4, respectively (Figure 4). The lowest AUDPC values were found in bunches treated with BIO+MPR3 (average value of 9.9 at the last monitoring time), thus confirming the good efficacy of the *M. pulcherrima* MPR3 strain in reducing powdery mildew disease. The disease progression over time was higher between the third and fourth monitoring time, except for the thesis BIO+MPR3 (in this case, the slope of curve increased starting from the second monitoring time).

**Figure 5:**
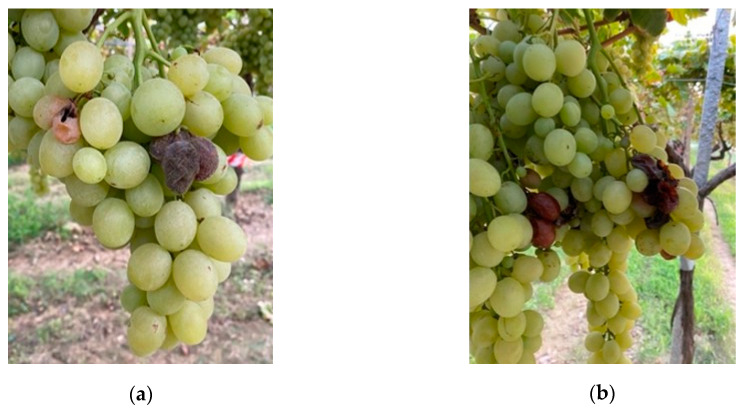
Symptoms observed on bunches of ‘Italia’ caused by *Botrytis cinerea*. Class of severity: 1 (1–25%) (**a**) and 2 (25.1–50%) (**b**).

The efficacy of *M. fructicola* NOLI and *M. pulcherrima* MPR3 against *B. cinerea* (Figure 5) was evaluated in the field from 2 September to 6 October 2022.

The ANOVA results obtained from datasets concerning grey mould decay were similar to those found for powdery mildew. As a matter of fact, disease parameters (DI, DS and I_MK_ values) were significantly affected by the single factors “treatment” and “monitoring time” (*p*-value ≤ 0.0001), whereas the effects of the factor “site” and of the interactions between factors were always not significant (Table 3). For this reason, once again, data from the two field trials were averaged and reported for each monitoring time.

Post hoc analyses highlighted significant differences among treatments for all disease parameters starting from the first monitoring time (Table 4). Albeit with different effectiveness, all treatments were able to reduce natural infections of *B. cinerea* on grape berries if compared to untreated control.

The results showed that the most effective treatment was once again BIO+MPR3, since it reduced the incidence of disease (DI) caused *B. cinerea* at the last monitoring time (6 October 2022) by approximately 68% if referred to untreated control (Table 4). Otherwise, treatments based on BIO, alone and in combination with NOLI, reduced the disease incidence by 30.4% and 37.5%, respectively, thus showing a similar efficacy. According to these results, both disease severity and the McKinney’s index were significantly lower on bunches treated with BIO+MPR3 (reductions of about 80%) than on bunches treated with BIO and BIO+NOLI (reductions from 43.1 to 51%) at the last monitoring time.

**Figure 6:**
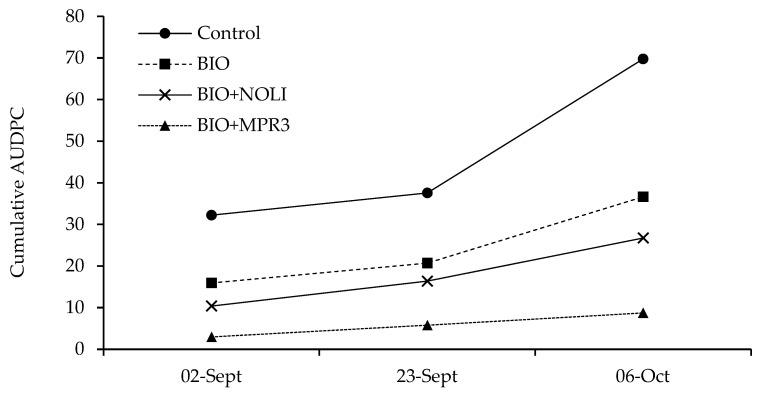
Area under disease progress curve (AUDPC) based on the McKinney’s index (%) of *Botrytis cinerea* on treated grapes with different formulations.

The progression over time of grey mould infections in the four different theses (control, BIO, BIO+NOLI and BIO+MPR3) was compared in the Figure 6. Comprehensively, in all of the theses, disease values reached their maximum level in October, at the last monitoring time. Moreover, the highest increase in grey mould was observed between the second and third monitoring time. The comparison of AUDPC values highlighted that disease increased during the season in a different manner according to treatment carried out on bunches, thus confirming the strong influence of treatments on disease parameters. In detail, the increase in disease was higher on the untreated control (final AUDPC average value of 69.9) and lower on bunches treated with BIO+MPR3, where it reached a final AUDPC average value of 8.7. On grape berries treated with BIO and BIO+NOLI, the final AUDPC values were equal to 36.6 and 26.7, respectively (Figure 6).

### 3.3. Effectiveness of Treatments against B. cinerea in Postharvest Environments

**Figure 7:**
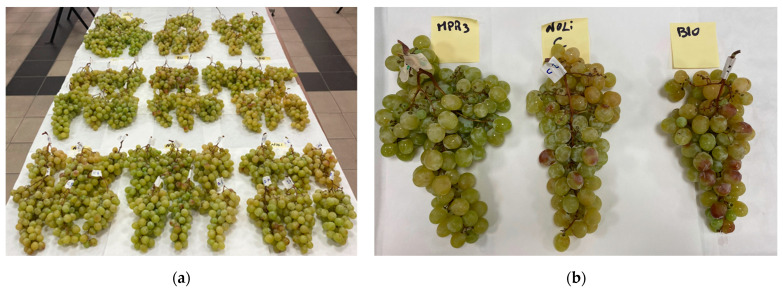
Bunches of table grape ‘Italia’ harvested from plants treated in the field with BIO (**top**), BIO+NOLI (**middle**) and BIO+MPR3 (**bottom**) treatments (**a**), and single grape clusters (**b**) after storage at 5 ± 1 °C for 10 days and 20 ± 1 °C for 4 days.

The effectiveness of field treatments based on *M. fructicola* strain NRRL Y-27328 (NOLI) and on *M. pulcherrima* MPR3 in controlling grey mould infections caused by *B. cinerea* was evaluated under postharvest conditions at two different times, i.e., at the end of the cold storage at 5 ± 1 °C for 10 days (first monitoring time, 14 October 2022) and at the end of the whole storage period, which included the storage of fruit at 20 ± 1 °C for the other 4 days (second monitoring time, 18 October 2022) (Figure 7).

As clearly shown in Table 5, disease incidence values did not change over time during the storage of fruits, whereas appreciable differences in the DS and I_MK_ values were found between the first and the second monitoring time, with greater severity values at the end of whole storage period. Field treatments based on yeasts, applied as a supplement to the BIO treatment, significantly reduced both DI and DS during the whole storage period if compared to the untreated control, whereas field treatments based on BIO were not able to protect grape berries from grey mould development, since no significant differences were found among the untreated control and the berries treated with BIO. At the end of the whole storage period (5 ± 1 °C for 10 days and 20 ± 1 °C for 4 days), the incidence and severity of disease were significantly lower in the bunches treated with BIO+MPR3 (DI and DS reduction of about 98%) than in those treated with BIO, alone and in combination with NOLI (DI and DS reductions ranging from 25.4 to 50.3%). Thus, preharvest applications of BIO+MPR3 showed the best effectiveness in controlling grey mould disease.

### 3.4. Evaluation of Epiphytic Microbial Population Associated to Grape Berries

Thirty-four days after the last treatment in the field, carpospheric fungal and bacterial populations, along with those of *Metschnikowia* spp., were separately evaluated for each of the four theses under study (control, BIO, BIO+NOLI and BIO+MPR3).

**Figure 8:**
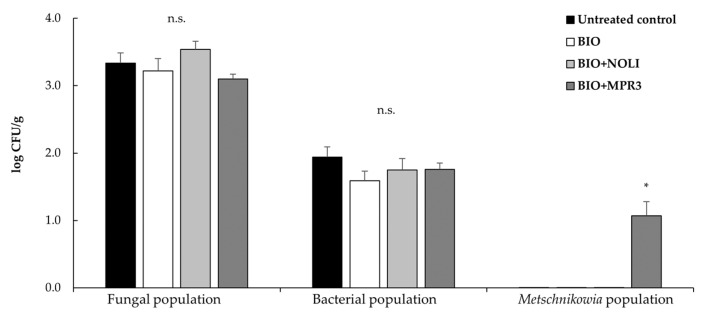
Fungal, bacterial and *Metschnikowia* spp. population on the grape’s carposphere after storage at 5 ± 1 °C for 10 days and 20 ± 1 °C for 4 days. Each value represents the mean of six replicates for treatment. Data are presented as Log_10_ CFU/g of fresh weight. Bars show the standard error of the mean (SEM). n.s.—not significant; * significant differences among treatments according to Fisher’s least significant difference test at *p*-value < 0.05.

The microbial load detected on the grape berries’ surface of each thesis is reported in Figure 8. The results highlighted that fungal and bacterial loads were not affected by the tested treatments if compared to the relatively untreated controls (no significant difference was observed among the four theses in the CFU/gr values detected for fungal and bacterial communities). The fungal load detected in grape berries ranged from 3.10 to 3.54 log CFU/g, while the bacterial load from 1.59 to 1.94 log CFU/g, throughout all treatments. In contrast, *Metschnikowia* spp. count was below the detection limit of viable plate count method (<10 CFU/g) in the untreated control grapes and in those treated with BIO and BIO+NOLI, while in the samples treated with BIO+MPR3, presumptive *Metschnikowia* spp. colonies were also detected. In this case, the *Metschnikowia* spp. load reached the value of 1.07 log CFU/g at the end of the whole storage period (Figure 8).

## 4. Discussion

The use of BCAs has gained an increased interest over time in integrated and organic strategies to control pre- and postharvest grape diseases. Several bacteria, filamentous fungi and yeasts have been assessed as potential antagonists through in vitro and in vivo evaluation of their antimicrobial activities. Among the growing array of effective biocontrol agents identified through research, antagonistic yeasts stand out particularly due to their inhibitory efficacy against numerous phytopathogenic fungi in the field and postharvest environments [26,27].

In the present study, we evaluated for the first time the antifungal activity of *M. pulcherrima* strain MPR3 and *M. fructicola* strain NRRL Y-27328 (commercial product NOLI) in combination with common applied organic treatments (BIO) under field conditions, specifically targeting grey mould and powdery mildew on table grape ‘Italia’, one of the most globally appreciated Sicilian cultivars. The open-field experiments provided compelling evidence of the excellent biocontrol efficacy of *M. pulcherrima* strain MPR3 against *B. cinerea*, as previously demonstrated in growth chamber experiments [13]. Specifically, the application of *M. pulcherrima* MPR3 three times during the 2022 summer season significantly reduced the incidence of powdery mildew caused by *E. necator*, as well as the incidence and severity of grey mould caused by *B. cinerea*, notably exhibiting higher efficacy compared to both BIO and BIO+NOLI treatments. While several studies have confirmed the effectiveness of biocontrol yeasts against *B. cinerea*, there is a lack of evidence regarding their activity against *E. necator*, which has been reported here for the first time. The observed efficacy of *M. pulcherrima* MPR3 against *E. necator* and *B. cinerea* in this study may be attributed to the competition for nutrients and space and to the production of secondary metabolites, such as pulcherrimin, which is recognized as one of the most active mechanisms of action for this species [22,28,29].

In general, the primary mechanism of action exerted by antagonistic yeasts involves the competition for nutrients and space [30]. The antagonists invade host fruits, exhibiting rapid growth, forming biofilms on the fruit surface and altering nutrient consumption [26], such as carbon and nitrogen, causing pathogenic fungi to face depletion and become unable to reach essential nutrients for their viability and growth [31]. Among these nutrients, iron can serve as a crucial limiting factor for fungal growth, as it is present in various proteins and enzyme structures. One of the most important mechanisms of action of antagonist yeasts is their ability to produce iron chelators and siderophores which compete for iron. This process results in iron depletion, subsequently inhibiting the conidial germination and pathogenesis of fungi [32]. By interfering with iron availability, the antagonists effectively obstruct the growth and development of pathogenic fungi, further contributing to their biocontrol activity. Pulcherriminic acid, which is secreted by certain antagonistic yeasts, plays a crucial role in iron chelation by reacting with Fe^3+^, and it produces an iron chelate known as pulcherrimin [29] and forms an insoluble red pigment whose intensity increases with the amount of depleting iron in the medium. The depletion of iron by pulcherrimin has been reported as responsible for inhibition of conidia germination, hyphal growth and lysis of hyphal tips of many phytopathogenic fungi such as *B. cinerea*, *A. alternata* and *P. expansum* [13,22,28]. Moreover, the iron depletion can be lethal or at least inhibitory to microorganisms that require higher amounts of iron for their cellular processes. Previous studies [13] have clearly indicated that multiple modes of action of *M. pulcherrima* MPR3 are responsible for its excellent control of several postharvest pathogens including botrytis rot on grape berries. The competition for iron through pulcherrimin production and the ability to form biofilm were hypothesized as the primary mechanisms of action for the MPR3 strain, along with volatile organic compound (VOC) production. As more recently evidenced [16], the antifungal activity of the MPR3 strain was confirmed to be strictly associated with its ability to compete for iron, also when exposed to *A. flavus* on pistachio nuts. This correlation is directly linked to the growth substrate, as the availability of nutrients is crucial in influencing the pathogenicity of *A. flavus* towards plants [33]. Hence, the yeast’s ability to compete with *A. flavus* for available iron through nutrient competition lays a solid foundation for the efficacy of the biocontrol strategy.

Furthermore, this study presents novel findings, revealing for the first time the long-lasting efficacy against grey mould following postharvest storage. In fact, even 20 days after the last field treatments and for the whole fruit storage period, both *M. pulcherrima* MPR3 and NOLI maintained their biocontrol efficacy. Little is known about the effects of the organic vineyard management on the postharvest control of *B. cinerea*; however, it is generally observed that the organic treatments do not provide effective long-term control of grey mould after cold storage. In this context, the present study demonstrates that the three-time field applications of BIO+MPR3 significantly reduced the incidence and severity of grey mould after 14 days of storage at low (5° C for 10 days) and room temperature (20 °C for 4 days). The efficacy of BIO+MPR3 was found to be higher than that of BIO and BIO+NOLI treatments, indicating its potential in protecting grape bunches during storage. Our results are consistent with a previous finding [25] where two preharvest applications of *M. pulcherrima* strain DiSVA269 in wine grapes effectively improved the postharvest control of *B. cinerea*. The involvement of indirect antagonistic mechanisms, such as the reduction of virulence of *B. cinerea* under iron-starvation conditions [22,34] or the stimulation of plant host defence pathways, as reported in strawberries treated with *M. fructicola* [35], may contribute to postharvest disease control. This hypothesis could potentially be extended to grapes as well.

The ability to control the disease maintaining the fruit quality under postharvest conditions is a crucial attribute for BCAs. In fact, postharvest conditions create an ideal environment for grey mould infection, as fruit tissues become less reactive due to weakening of defence mechanisms after harvest and during cold storage. The ability of *Metschnikowia* species to survive at low temperatures is a significant biocontrol feature, particularly since table grapes are commonly stored at low temperatures during postharvest to extend their shelf life and availability. Previous studies observed the ability of *M. pulcherrima* to survive and even grow at low temperatures [25,36]. The low survival ability of *Metschnikowia* spp. on grape berries observed in this study could be attributed to the long interval between the last treatment in the field (14 September) and the date when the yeast population was monitored (18 October). Despite the observed modest survival capacity, *Metschnikowia* species were still able to maintain their efficacy in reducing disease parameters during postharvest storage, having no negative impact on native carpospheric microbial communities. This highlights the promising role of *Metschnikowia* strains as a biocontrol agent in organic vineyard management and postharvest disease control, providing a valuable contribution to sustainable viticulture practices. The results presented here, along with the supporting evidence from previous studies, emphasize the potential of antagonistic yeasts, particularly of the MPR3 strain, as promising tools in the integrated approach in managing grape pathogens and enhancing the quality and shelf life of grape products. Further investigations into the underlying mechanisms and optimization of application strategies would be valuable in fully harnessing the benefits of these biocontrol agents for the grape industry.

## 5. Conclusions

*M. pulcherrima* strain MPR3 combined with organic control measures could be proposed to enhance the sustainability of the organic management of the main airborne diseases of table grapes. These efforts could hopefully allow a significant reduction over time in the use of copper in organic viticulture according to global green policies. Nevertheless, further studies are needed to confirm the antagonistic activity of *M. pulcherrima* strain MPR3 applied in the field on different biological cultures and against different pathogens and to verify how the field conditions, such as temperature swings, high humidity, water, solar radiation and interaction with the resident microbiota could affect yeast viability and biocontrol efficacy during the postharvest stage.

## Figures and Tables

**Table 1:** Effects of single factors and their interactions in ANOVA on the powdery mildew infection on table grapes caused by *Erysiphe necator* over time.

		Disease Incidence (DI)		Disease Severity (DS)		McKinney’s Index (I_MK_)	
Source of Variation	df	F	*p*-Value	F	*p*-Value	F	*p*-Value
Time	3	4.60	0.003	8.53	<0.0001	*6.95*	<0.0001
Site	1	2.11	0.147 ^ns^	2.69	0.102 ^ns^	2.12	0.146 ^ns^
Treatment	3	27.22	<0.0001	22.38	<0.0001	26.92	<0.0001
Time × treatment	9	0.50	0.873 ^ns^	0.85	0.571 ^ns^	0.69	0.716 ^ns^
Treatment × site	3	0.37	0.778 ^ns^	0.55	0.650 ^ns^	0.37	0.772 ^ns^
Treatment × time × site	9	0.11	1.000 ^ns^	0.16	0.997 ^ns^	0.09	1.000 ^ns^

F—test of fixed effects; df—degrees of freedom and *p*-value associated to F; ns—non-significant data.

**Table 2:** Post hoc analyses of treatment effects on disease incidence (DI), severity (DS) and McKinney’s index (I_MK_) of powdery mildew caused by *Erysiphe necator* over time on ‘Italia’ table-grape bunches.

	30 July 2022			16 August 2022			2 September 2022			23 September 2022		
Treatment	DI (%) ^a,b^	DS (0–3) ^a^	I_MK_ (%) ^a,b^	DI (%) ^a,b^	DS (0–3) ^a^	I_MK_ (%) ^a,b^	DI (%) ^a,b^	DS (0–3) ^a^	I_MK_ (%) ^a,b^	DI (%) ^a,b^	DS (0–3) ^a^	I_MK_ (%) ^a,b^
Control	1.667 ± 0.492 a	0.019 ± 0.006 a	0.629 ± 0.198 a	2.556 ± 0.506 a	0.029 ± 0.006 a	0.963 ± 0.203 a	2.778 ± 0.540 a	0.036 ± 0.008 a	1.185 ± 0.256 a	3.222 ± 0.563 a	0.049 ± 0.009 a	1.630 ± 0.306 a
BIO	0.667 ± 0.362 ab	0.007 ± 0.004 b	0.222 ± 0.121 b	1.444 ± 0.506 b	0.017 ± 0.007 ab	0.556 ± 0.217 ab	1.556 ± 0.473 b	0.019 ± 0.006 b	0.630 ± 0.205 b	1.667 ± 0.492 b	0.028 ± 0.009 b	0.926 ± 0.296 b
BIO+NOLI	0.889 ± 0.403 b	0.009 ± 0.004 b	0.296 ± 0.134 b	0.111 ± 0.403 bc	0.011 ± 0.004 bc	0.370 ± 0.134 bc	1.222 ± 0.432 bc	0.013 ± 0.005 bc	0.444 ± 0.152 bc	1.556 ± 0.473 b	0.016 ± 0.005 bc	0.519 ± 0.158 bc
BIO+MP3	0.222 ± 0.222 b	0.002 ± 0.002 b	0.074 ± 0.074 b	0.333 ± 0.243 c	0.003 ± 0.002 c	0.111 ± 0.081 c	0.444 ± 0.305 c	0.004 ± 0.003 c	0.148 ± 0.102 c	0.444 ± 0.258 c	0.013 ± 0.008 c	0.444 ± 0.258 c

^a^ Data expressed are means of the two trials, followed by standard error of the means (±SEM). Each value derives from 36 plants (18 plants for each experimental trial), each formed by 25 bunches. ^b^ Arcsine transformation was used on percentage data prior to analysis, whereas back-transformed estimates (%) are presented. DI, DS and I_MK_ values followed by different letters in the same column are significantly different according to Fisher’s least significant difference test at *p*-value < 0.05.

**Table 3:** Effects of single factors and their interactions in ANOVA on the grey mould infection on table grapes caused by *Botrytis cinerea* over time.

		Disease Incidence (DI)		Disease Severity (DS)		McKinney’s Index (I_MK_)	
Source of Variation	df	F	*p*-Value	F	*p*-Value	F	*p*-Value
Time	3	42.3	<0.0001	23.95	<0.0001	38.02	<0.0001
Site	1	0	0.95 ^ns^	0.03	0.86 ^ns^	0.07	0.79 ^ns^
Treatment	3	23.06	<0.0001	18.52	<0.0001	25.98	<0.0001
Time × treatment	9	1.02	0.41 ^ns^	1.7	0.12 ^ns^	1.49	0.18 ^ns^
Treatment × site	3	0.49	0.69 ^ns^	0.71	0.55 ^ns^	0.78	0.50 ^ns^
Treatment × time × site	9	0.16	0.98 ^ns^	0.34	0.91 ^ns^	0.24	0.96 ^ns^

F—test of fixed effects; df—degrees of freedom and p-value associated to F; ns—non-significant data.

**Table 4:** Post hoc analyses of treatment effects on disease incidence (DI), severity (DS) and McKinney’s index (I_MK_) of grey mould caused by *Botrytis cinerea* over time on table grape ‘Italia’ bunches.

	2 September 2022			23 September 2022			4 October 2022		
Treatment	DI (%) ^a,b^	DS (0–3) ^a^	I_MK_ (%) ^a,b^	DI (%) ^a,b^	DS (0–3) ^a^	I_MK_ (%) ^a,b^	DI (%) ^a,b^	DS (0–3) ^a^	I_MK_ (%) ^a,b^
Control	1.889 ± 0.498 a	0.026 ± 0.007 a	0.852 ± 0.240 a	4.111 ± 0.636 a	0.071 ± 0.016 a	2.370 ± 0.540 a	6.222 ± 0.540 a	0.102 ± 0.018 a	3.407 ± 0.607 a
BIO	0.778 ± 0.367 b	0.010 ± 0.005 b	0.333 ± 0.155 b	2.333 ± 0.691 b	0.038 ± 0.013 b	1.259 ± 0.437 b	4.333 ± 0.904 b	0.058 ± 0.014 b	1.926 ± 0.466 b
BIO+NOLI	0.556 ± 0.315 b	0.006 ± 0.003 b	0.185 ± 0.105 b	2.111 ± 0.471 b	0.026 ± 0.006 bc	0.852 ± 0.214 b	3.889 ± 0.921 b	0.050 ± 0.015 b	1.667 ± 0.506 b
BIO+MP3	0.222 ± 0.222 b	0.002 ± 0.002 b	0.074 ± 0.074 b	0.667 ± 0.485 c	0.007 ± 0.005 c	0.222 ± 0.162 c	2.000 ± 0.741 c	0.020 ± 0.007 c	0.667 ± 0.247 c

^a^ Data expressed are means of the two trials, followed by standard error of the means (±SEM). Each value derives from 36 plants (18 plants for each experimental trial), each formed by 25 bunches. ^b^ Arcsine transformation was used on percentage data prior to analysis, whereas back-transformed estimates (%) are presented. DI, DS and I_MK_ values followed by different letters in the same column are significantly different according to Fisher’s least significant difference test at *p*-value < 0.05.

**Table 5:** Disease incidence (DI), disease severity (DS) and McKinney’s index (I_MK_) of grey mould caused by *Botrytis cinerea* on ‘Italia’ table grapes, treated in the field with different formulations and, subsequently, kept at 5 ± 1 °C for 10 days (14/10/2022) and at 20 ± 1 °C for 4 days (18/10/2023). Disease parameters were evaluated at the end of the two storage periods.

	14 October 2022			18 October 2022		
Treatment	DI (%) ^a,b^	DS (0–3) ^a^	I_MK_ (%) ^a,b^	DI (%) ^a,b^	DS (0–3) ^a^	I_MK_ (%) ^a,b^
Control	6.71 ± 1.33 a	0.112 ± 0.025 a	3.74 ± 0.84 a	6.95 ± 1.30 a	0.167 ± 0.034 a	5.57 ± 1.15 a
BIO	5.18 ± 1.41 ab	0.083 ± 0.026 ab	2.76 ± 0.85 ab	5.18 ± 1.41 ab	0.123 ± 0.034 ab	4.10 ± 1.12 ab
BIO+NOLI	3.55 ± 1.00 b	0.051 ± 0.017 b	1.70 ± 0.58 b	3.55 ± 1.00 b	0.083 ± 0.025 b	2.77 ± 0.83 b
BIO+MP3	0.08 ± 0.08 c	0.001 ± 0.001 c	0.03 ± 0.03 c	0.08 ± 0.08 c	0.002 ± 0.002 c	0.01 ± 0.01 c

^a^ Data are means of three replicates, each formed by 6 bunches (18 bunches/thesis), followed by standard error of the means ± SEM. ^b^ Arcsine transformation was used on percentage data prior to analysis, whereas back-transformed estimates (%) are presented. Values followed by different letters in the same column are significantly different according to Fisher’s least significant difference test at *p*-value < 0.05.

## Data Availability

The data used to support the findings of this study can be made available by the corresponding author upon request.
